# Association between self-care disability and depressive symptoms among middle-aged and elderly Chinese people

**DOI:** 10.1371/journal.pone.0266950

**Published:** 2022-04-11

**Authors:** Ting-Yu Mu, Ri-Xiang Xu, Jia-Yi Xu, Die Dong, Zhi-Nan Zhou, Jia-Ning Dai, Cui-Zhen Shen

**Affiliations:** 1 School of Nursing, Zhejiang Chinese Medical University, Hangzhou, Zhejiang Province, China; 2 Nursing College, Anhui University of Chinese Medicine, Hefei, Anhui Province, China; 3 School of Humanities and Management, Zhejiang Chinese Medical University, Hangzhou, Zhejiang Province, China; Ritsumeikan University, JAPAN

## Abstract

**Objective:**

In the context of an increased focus on geriatric depression in recent years, this study examined the associations between different types of self-care disability, the number of self-care disabilities, and depressive symptoms among middle-aged and elderly Chinese people.

**Method:**

The data for this study were extracted from the follow-up survey (conducted in 2018) of the China Health and Retirement Longitudinal Study (CHARLS). The sample comprised 10808 participants aged 45 years and older. The Activities of Daily Living (ADL) scale and the Center for Epidemiological Studies Depression (CESD-10) Scale were used to assess self-care disability and depressive symptoms, respectively.

**Result:**

The prevalence of depressive symptoms and self-care disability among the surveyed residents was 45.1% and 23.4%, respectively. Overall, there was a significant positive association between self-care disability and depressive symptoms. Participants who reported having a self-care disability in relation dressing, bathing, transferring in and out of bed, using the toilet, and controlling urination and defecation were found to have a significantly higher risk of depressive symptoms. In addition, participants with a greater cumulative quantity of self-care disabilities had a higher risk of depressive symptoms, and higher CESD-10 scores.

**Conclusion:**

Self-care disability is a risk factor for depressive symptoms among middle-aged and elderly Chinese people. A positive correlation between the number of self-care disabilities and the risk of depressive symptoms was found.

## Introduction

Depression is a common mental disease worldwide that negatively affects patients’ psychosocial functioning and quality of life [1]. Depression comprises three major depressive symptoms, including emotional symptoms, neurovegetative symptoms, and neurocognitive symptoms, depressed mood and anhedonia are the fundamental symptoms of depression [1]. When depressive symptoms are present, but not numerous or severe enough to be considered a syndrome, they are sometimes referred to as subthreshold depressive symptoms [1]. Depressive symptoms will gradually develop into depression if the severity of these symptoms cannot be improved over time [2]. Therefore, depressive symptoms are important because they can serve as early indicators of a major depressive episode [1]. The lifetime and 12-month prevalence of depression are estimated to be 15–18% and 7.2%, respectively [3, 4]. Meanwhile, in primary care, 1 in 10 patients, on average, present with depressive symptoms [5]. Globally, the prevalence of depression peaks in older adulthood among those aged 55–74 years [6]. The high prevalence of depression increases the morbidity and risk of other comorbid diseases, leading to greater health expenditure. Depression accounts for 3.8% of the total all-cause disability-adjusted life years globally [7], and is predicted to be one of the most significant causes of disease burden in middle and higher-income countries by 2030 [8]. Moreover, in the absence of scaled-up treatment, depression and anxiety will result in a loss of productivity equivalent to $1.15 trillion every year [9]. However, according to data from the World Health Organization (WHO), the prevalence of treatment for depression was only 0.096% in 2017, and 0.018% in low-income countries [10]. This highlights the need for a research focus on the treatment of depression and depressive symptoms.

Disability is an increasing public health issue, defined as a series of life obstacles that impact an individual’s physical, social, recreational, and employment activities [11]. Compared with depression, disability causes a more serious loss of quality of life and a greater economic burden, as some individuals with severe physical disabilities require lifelong care. The causes of disability are complex and multifaceted and include non-communicable and communicable diseases as well as injuries. Commonly, disability is measured using the Activities of Daily Living (ADL) scale or the Instrument Activities of Daily Living (IADL) scale [12]. In the current study, self-care disability was measured using the ADL scale, which reflects an individual’s need for assistance with daily self-care, such as bathing, eating, and dressing.

There is a robust association between depression and disability [13, 14]. Previous studies have suggested that the relationship between the two might be reciprocal [11, 15, 16]. Specifically, depression is associated with decreased motivation, which leads to reduced self-care, poor nutrition, and lack of exercise, which can further worsen disability [17]. Conversely, disability can increase the risk of depression due to poor social relationships [18]. Fan et al. found that ADL disability influences depressive symptoms in middle-aged and elderly Chinese people [19, 20]. However, not all studies support this view. A multi-center cohort study found that an initial change in depression was associated with subsequent change in disability, but a change in disability was not related to increased depression [21]. In addition, the prevalence of depressive symptoms is influenced by demographics (such as gender, age, marital status, cultural background, income level, education level, hukou) and morbidity of chronic diseases[19, 22, 23].

Differential trends in the prevalence of disability have been reported for different disability types [24], and different disability types have different risks of depressive symptoms [11]. Xiang suggested that more studies should confirm the differential associations between depressive symptoms and different types of disability [11]. However, to date, research on middle-aged and elderly people in China has focused on the influence of disability compared to no disability, the degree of disability, or depressive symptoms among the elderly living in rural areas [19, 25, 26], while ignoring the influence of different types of disabilities on depressive symptoms. Of note, ADL refers to the most basic and common body movements that people must perform repeatedly in their daily lives when living independently, such as eating, dressing, bathing, transferring in and out of bed, using the toilet, and controlling urination and defecation; these activities reflect the most basic self-care abilities [27]. Therefore, given the aging population in China, this study focused on self-care disability, and aimed to explain the relationships between different types of self-care disability, number of self-care disabilities, and depressive symptoms among Chinese adults aged ≥45 years using data from the China Health and Retirement Longitudinal Study (CHARLS).

## Methods

### Sample

**Fig 1:**
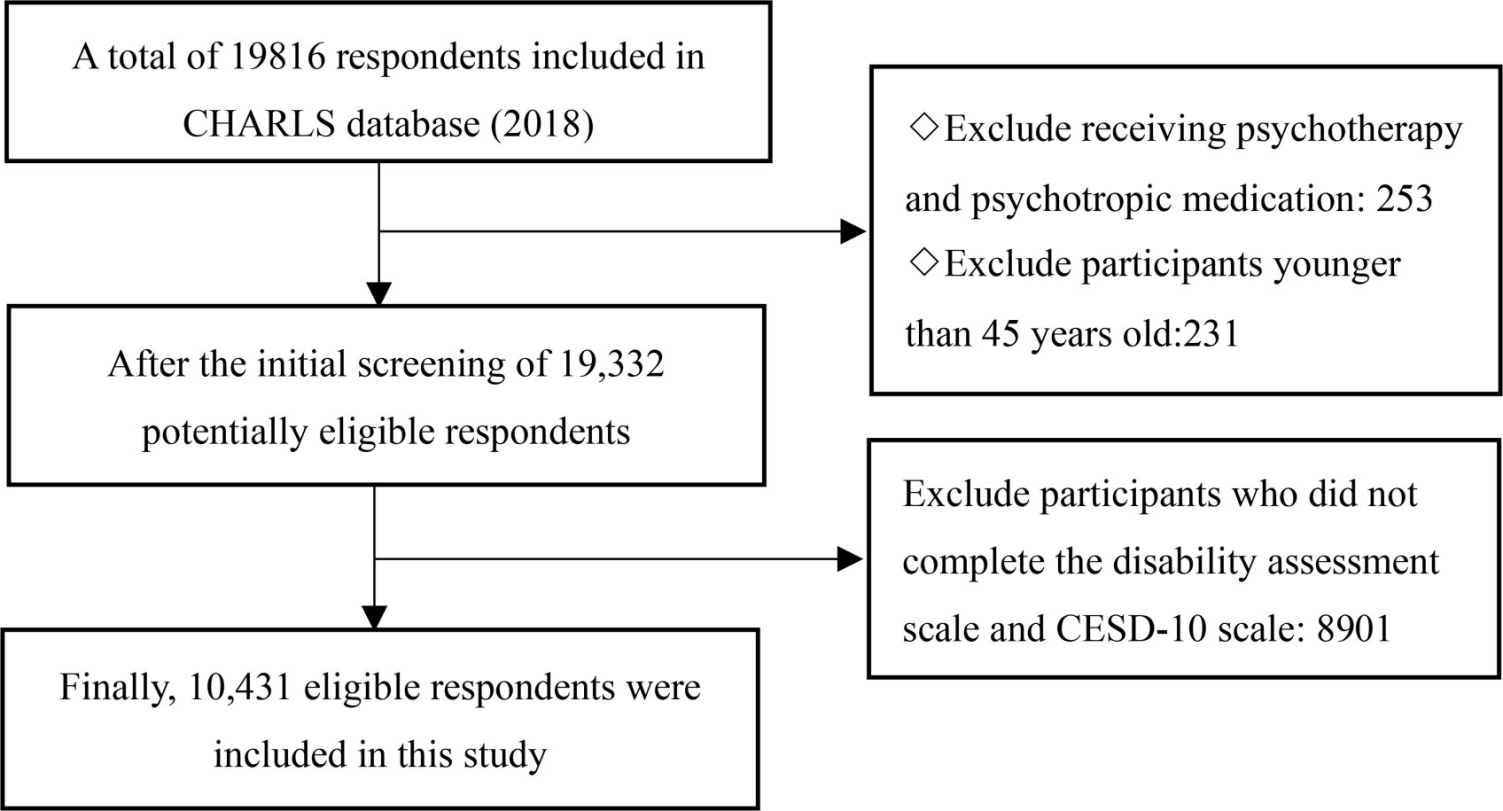
Flowchart of subject recruitment and eligibility.

The data analyzed in this study were obtained from a nationally representative cohort survey, the CHARLS, which was conducted by the China Social Science Survey Centre at Peking University. The CHARLS is an open-access public database for all researchers. It aims to measure the health status, economic status, and well-being of Chinese residents aged ≥45 years. It is updated every two-three years, with a total of four waves currently available (2011, 2013, 2015, 2018). This study used data from the latest waves, with the data collected between July 2018 and March 2019. The data utilized in this study included demographic data and data on the health condition of residents. The original dataset included data for 19744 respondents. In the current study, respondents were excluded if: they were aged less than 45 years, had missing scores for the 10-item Center for Epidemiological Studies Depression Scale (CESD-10), were receiving psychiatric or psychological treatment, and/or were taking psychotropic drugs. After the application of the exclusion criteria, a total of 10431 eligible respondents were included in this study. The screening process to determine eligibility is shown in Fig 1. All participants provided written informed consent at the time of participation and the Biomedical Ethics Review Committee of Peking University approved the CHARLS data collection (IRB00001052–11015).

### Measures

#### Depressive symptoms

The CESD-10 scale was selected as the measure of depressive symptoms in this survey. This scale has adequate validity and reliability for the general Chinese population [28, 29]. The scale contains 10 questions that measure the frequency of the respondent’s negative feelings in the past week. Each question has four response options: rarely or none of the time (0), some or a little of the time (1), occasionally or a moderate amount of the time (2), and most or all of the time (3). The total score ranges from 0 to 30, with scores ≥ 10 indicating significant depressive symptoms. This measure has high sensitivity and specificity [30].

#### Self-care disability

The ADL scale was used to estimate each respondent’s self-care disability [31]. The scale comprises six items that measure the respondent’s level of difficulty with performing self-care activities. Self-care disability was identified if the respondent answered “have difficulty to do” or “cannot do at all/unable to do” to one of the following six self-care activities: eating, dressing, bathing, transferring in and out of bed, using the toilet, and controlling urination and defecation [32].

#### Other measures (covariates)

Various control variables were included in the analyses in this study, including demographic characteristics (such as gender, age, location of residence, educational level, marital status, income and hukou status) and health status (such as mental or emotional problems, memory disorder and chronic diseases). These covariates were included in this study based on previous studies or preliminary univariate analyses indicating that they were confounders for depressive symptoms. Age was classified as 60 years and above or below 60 years (60 years is the retirement age in China). Hukou status (hukou is a centuries-old Chinese word referring to the national household registration that Chinese governments have historically used to try to fix the population in place geographically) was divided into agricultural and non- agricultural. Marital status was categorized as married with spouse present, never married, widowed, separated, or divorced. Educational level was categorized as illiterate, elementary school and below, middle school, high school/vocational school, or college degree and above. A total of 12 chronic diseases were included, namely hypertension, dyslipidemia, diabetes, cancer, chronic lung diseases, liver disease, heart attack, stroke, kidney disease, digestive disease, arthritis /rheumatism and asthma. Income was classified according to the classification of different economies by the World Bank in 2018 (low-income households were those with an annual income of $995 or less; lower middle-income households were those with an annual income between $996 and $3,895; upper middle-income households were those with an annual income between $3,896 and $12,055; high-income households were those with an annual income of $12,056 or more). In addition, mental/emotional problems and memory disorders were considered as potential confounders.

### Data analyses

The original data from the CHARLS database were exported in DTA format to Microsoft EXCEL 2016 for data screening and description. Statistical analysis was performed using SPSS 25.0 (SPSS Inc., Chicago, IL, US). The chi-squared test was used for comparison of the baseline characteristics between the depressive symptoms group and the non-depressive symptoms group. The Enter algorithm was used in the multivariate analysis models to assess the net effect of each potential factor. A linear regression model adjusting for all significant univariate factors was conducted to evaluate the association between CESD-10 scores and self-care disability. The results are expressed as regression coefficients (B values) and 95% confidential intervals (CIs). Binary logistic regression was used to examine the association between depressive symptoms and self-care disability after adjusting for possible confounding factors. *P* values less than 0.05 were considered statistically significant.

## Results

### Sample characteristics

A total of 10431 eligible respondents were included in this study, with a mean age of 62.37±9.36 years. The majority of participants were female (57.9%), had an agricultural hukou (79.4%), had a relatively low education level (illiterate or elementary and below education; 68.9%), and were married with a spouse present (79.3%). In total, 45.1% of respondents had depressive symptoms, with a mean CESD-10 score of 9.73±6.70, and 23.4% had self-care disability. More details of the sample characteristics are presented in **Table 1**.

**Table 1:** Sample characteristics of middle- and older-aged Chinese respondents.

Characteristics	Total (%)	No depressive symptoms (5728)	Depressive symptoms (4703)	*χ* ^*2*^	*P*
**Gender**				111.74	<0.001
Male	4395(42.13)	2678	1717		
Female	6036(57.87)	3050	2986		
**Age**				4.85	0.028
<60	4135(40.23)	2215	1920		
≥60	6143(59.77)	3426	2717		
**location of residence**				137.15	<0.001
Rural	7560(72.70)	3882	3678		
Urban	2839(27.30)	1822	1017		
**Education level**				185.53	<0.001
Illiterate	2359(22.62)	1117	1242		
Elementary school and below	4824(46.25)	2548	2276		
Middle school	2150(20.61)	1300	850		
High school/vocational School	951(9.12)	654	297		
College degree and above	147(1.41)	109	38		
**Marital status**				62.39	<0.001
Married with spouse present	8268(79.26)	4700	3568		
Never Married	49(0.47)	21	28		
Widowed	1315(12.61)	618	697		
Separated	681(6.53)	326	355		
Divorced	118(1.13)	63	55		
**Hukou status (household registration)**				135.35	<0.001
Agricultural	8153(79.43)	4235	3918		
Non-agricultural	2111(20.57)	1395	716		
**Self-care disability**				507.77	<0.001
Yes	2446(23.45)	1588	858		
No	7985(79.55)	3115	4870		
**Mental or emotional problems**				13.66	<0.001
Yes	58(0.56)	18	40		
No	10289(99.44)	5684	4605		
**Memory disorders**				32.08	<0.001
Yes	207(2.02)	74	133		
No	10028(97.98)	5569	4459		
**Number of chronic conditions**				102.70	<0.001
0	5337(51.16)	3158	2179		
1	3110(29.81)	1646	1464		
2	1230(11.79)	591	639		
3	463(4.44)	206	257		
≥4	291(2.79)	127	164		
**Annual households’ income**				258.82	<0.001
High-income	1085(10.40)	752	333		
Upper middle-income	3120(29.91)	1930	1190		
Lower middle-income	2563(24.57)	1318	1245		
Low-income	3663(35.12)	1728	1935		

### Associations between self-care disability and depressive symptoms

The results of the univariate analyses are presented in **Table 2**. Female participants (OR = 1.53, 95%CI = 1.41–1.65), those 60 years or older (OR = 1.09, 95%CI = 1.01–1.18), those living in a rural area (OR = 1.70, 95%CI = 1.55–1.86), those with an agricultural hukou (OR = 1.80, 95%CI = 1.63–1.99), those with mental or emotional problems (OR = 2.74, 95%CI = 1.57–4.79), those with memory disorders (OR = 2.24, 95%CI = 1.68–2.99), and those with self-care disability (OR = 2.89, 95%CI = 2.63–3.18) had a significantly higher risk of depressive symptoms (*P*<0.05). In addition, participants with different education levels, household incomes, number of chronic diseases and marital status had different risks of depressive symptoms (*P*<0.001).

**Table 2:** Crude and adjusted odds ratios (OR) for the associations between self-care disability and depressive symptoms.

Characteristics	Unadjusted		*P*-value	Adjusted^a^		*P*-value
	OR	95%CI		OR	95%CI	
**Gender (ref: male)**	1.53	1.41–1.65	<0.001	1.43	1.30–1.57	<0.001
**Age (ref:≥60)**	1.09	1.01–1.18	0.028	1.36	1.24–1.49	<0.001
**location of residence (ref: urban)**	1.70	1.55–1.86	<0.001	1.16	1.02–1.32	0.022
**Education level(ref:College degree and above)**						
Illiterate	3.19	2.19–4.65	<0.001	1.32	0.86–2.03	0.200
Primary school and below	2.56	1.76–3.72	<0.001	1.26	0.83–1.90	0.285
Middle school	1.88	1.28–2.74	0.001	1.06	0.70–1.61	0.774
High school/vocational School	1.30	0.88–1.93	0.190	0.85	0.56–1.31	0.459
**Marital status (ref: married with spouse present)**						
Never Married	1.76	1.00–3.10	0.049	1.36	0.72–2.55	0.342
Widowed	1.49	1.30–1.67	<0.001	1.21	1.06–1.38	0.005
Separated	1.43	1.23–1.68	<0.001	1.37	1.15–1.62	<0.001
Divorced	1.15	0.80–1.66	0.452	1.16	0.77–1.73	0.482
**Hukou status (ref: non-agriculture)**	1.80	1.63–1.99	<0.001	1.16	1.01–1.34	0.043
**Mental or emotional problems (ref:no)**	2.74	1.57–4.79	<0.001	1.47	0.78–2.77	0.230
**Memory disorders (ref:no)**	2.24	1.68–2.99	<0.001	1.97	1.44–2.71	<0.001
**Disability(ref: no)**	2.89	2.63–3.18	<0.001	2.60	2.34–2.88	<0.001
**Number of chronic conditions(ref:0)**						
1	1.29	1.18–1.41	<0.001	1.24	1.12–1.36	<0.001
2	1.57	1.38–1.78	<0.001	1.55	1.35–1.77	<0.001
3	1.81	1.49–2.19	<0.001	1.75	1.42–2.15	<0.001
≥4	1.87	1.48–2.37	<0.001	1.86	1.42–2.43	<0.001
**Households annual income(ref: high-income)**						
Upper middle-income	1.39	1.20–1.61	<0.001	1.20	1.02–1.40	0.030
Lower middle-income	2.13	1.84–2.48	<0.001	1.61	1.36–1.91	<0.001
Low-income	2.53	2.19–2.92	<0.001	1.86	1.57–2.20	<0.001

a: Adjusted for the all confounding factors which are shown in Table 1

The result of the binary logistic regression analysis testing the association between self-care disability and depressive symptoms is shown in **Table 2**. The risk of depressive symptoms among respondents with self-care disability was significantly higher than that among respondents without self-care disability (aOR = 2.60, 95%CI = 2.34–2.88, *P*<0.001). In addition, significant associations between gender, age, location of residence, marital status, hukou status, memory disorders, income, number of chronic diseases, and depressive symptoms were observed, consistent with the results of the univariate analyses. However, there were no significant associations between depressive symptoms and both education level and mental or emotional problems in the adjusted analysis (*P*>0.05).

**Fig 2:**
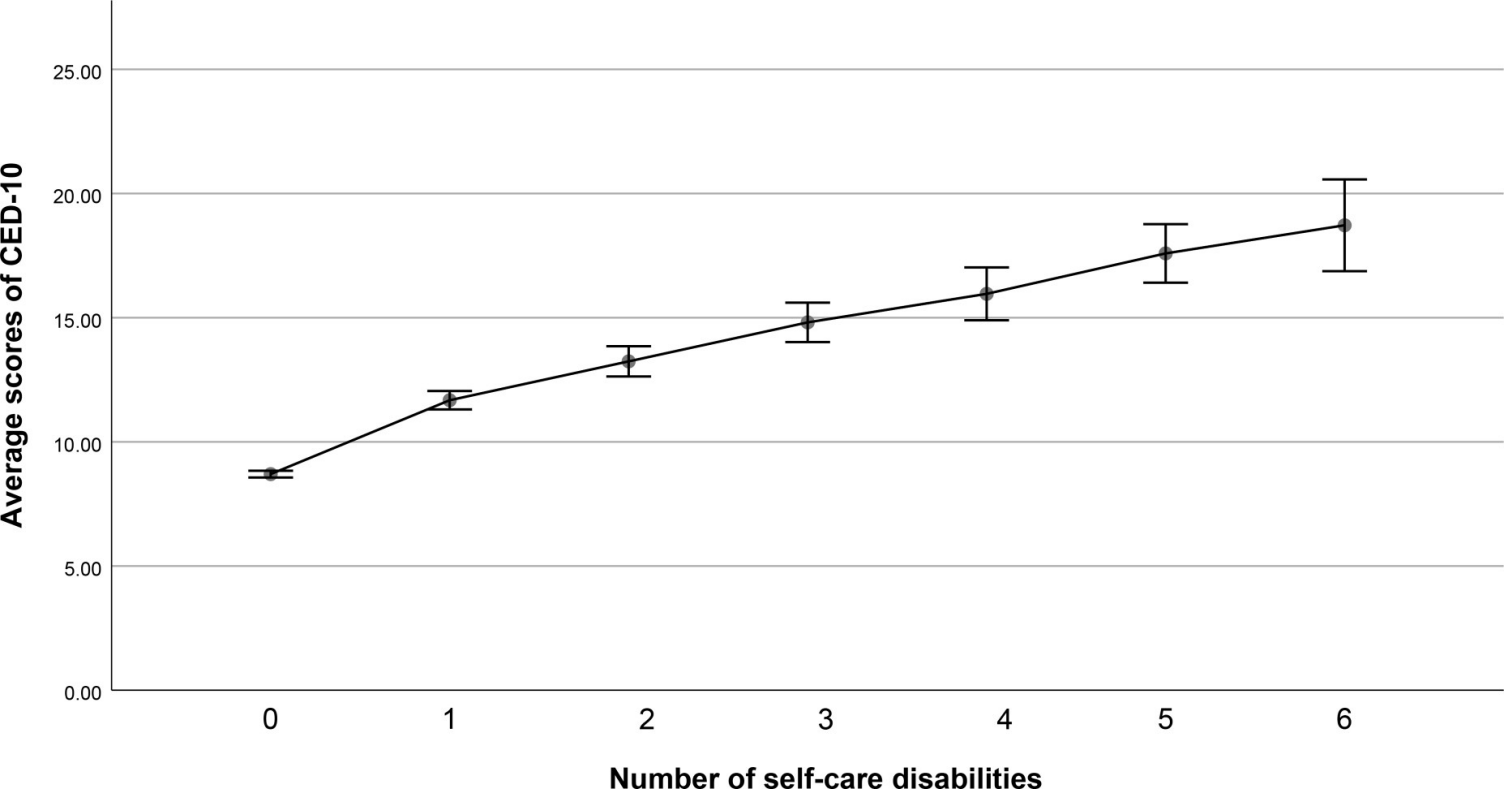
The relationship between the number of self-care disabilities and CESD-10 scores.

The associations between types of self-care disability and depressive symptoms were also analyzed using a binary logistic regression model. The results suggested that respondents with a disability related to dressing, bathing, transferring in and out of bed, eating, using the toilet, and controlling urination and defecation had a significantly higher risk of depressive symptoms than those without these disabilities, with aORs of 1.56 (95%CI: 1.27–1.92, *P*<0.001), 1.81 (95%CI: 1.49–2.19, *P*<0.001), 1.49 (95%CI: 1.22–1.83, *P*<0.001), 1.53 (95%CI: 1.33–1.76, *P*<0.001), and 2.26 (95%CI: 1.80–2.85, *P*<0.001), respectively (**Table 3**). In addition, the quantity of self-care disabilities was analyzed to evaluate the association between the number of self-care disabilities and depressive symptoms. In the adjusted model, when the participants had any quantity of self-care disability, the risk of depressive symptoms was significantly higher, as compared to respondents without disability (**Table 4**). Further, those with a greater quantity of self-care disability had a higher risk of depressive symptoms, and a disability quantity equaling 6 had the highest risk depressive symptoms (aOR = 13.70, 95%CI = 4.86–38.58, *P*<0.001). Moreover, **Fig 2** shows that there was a positive correlation between the number of self-care disabilities and CESD-10 scores (B = 1.97, SE = 0.06, *P*<0.001).

**Table 3:** Crude and adjusted odds ratios (OR) for the associations between types of self-care disability and depressive symptoms.

Type of self-care disability	Depressive symptoms	No depressive symptoms	Unadjusted		*P*	Adjusted^a^		*P*
			OR	95%CI		OR	95%CI	
① Difficulty in dressing	573	213	3.59	3.05–4.23	<0.001	1.56	1.27–1.92	<0.001
② Difficulty in bathing	677	247	3.73	3.21–4.34	<0.001	1.81	1.49–2.19	<0.001
③ Difficulty in eating	189	52	4.57	3.35–6.23	<0.001	1.38	0.95–2.02	0.094
④ Difficulty in transferring in and out of bed	584	211	3.71	3.15–4.36	<0.001	1.49	1.22–1.83	<0.001
⑤ Difficulty in using the toilet	982	492	2.81	2.50–3.15	<0.001	1.53	1.33–1.76	<0.001
⑥ Difficulty in controlling urination and defecation	398	133	3.89	3.18–4.75	<0.001	2.26	1.80–2.85	<0.001

a: Adjusted for the possible confounding factors which are shown in Table 1

**Table 4:** Crude and adjusted odds ratios (OR) for the associations between quantity of self-care disability and depressive symptoms.

Number of disabilities	No depressive symptoms	Depressive symptoms	Unadjusted		*P*	Adjusted^a^		*P*
			OR	95%CI		OR	(95%CI)	
0	4870	3115	ref.	ref.	*/*	ref.	ref.	*/*
1	557	742	2.08	1.85–2.35	<0.001	1.92	1.69–2.18	<0.001
2	188	352	2.93	2.44–3.51	<0.001	2.65	2.18–3.23	<0.001
3	60	210	5.47	4.09–7.32	<0.001	5.10	3.73–6.98	<0.001
4	35	138	6.16	4.24–8.96	<0.001	5.03	3.40–7.43	<0.001
5	13	101	12.15	6.81–21.68	<0.001	10.07	5.43–18.68	<0.001
6	5	45	14.07	5.58–35.49	<0.001	13.70	4.86–38.58	<0.001

a: Adjusted for the possible confounding factors which are shown in Table 1

## Discussion

Self-care disability affects the daily lives and social functioning of the middle-aged and elderly. This is especially important today given the continual aging of China’s population. Previous studies have reported a reciprocal relationship between physical and mental health in older adults, highlighting the need to pay attention to the mental health of older people with physical health problems [33]. In the present study, using data from a large public database of Chinese middle-aged and elderly people, the association between self-care disability and depressive symptoms was explored and the possible influencing factors were evaluated. The prevalence of depressive symptoms was 45.1% and the prevalence of self-care disability was 23.45%. Compared to people without self-care disabilities, those with a self-care disability had a higher risk of depressive symptoms, particularly for those who reported requiring assistance with dressing, eating, and controlling urination and defecation. Moreover, there was a positive correlation between the number of self-care disabilities and the risk of depressive symptoms. In addition to the relationship between self-care disability and depressive symptoms, relationships between depressive symptoms and gender, age, location of residence, education level, marital status, hukou status, mental or emotional problems, memory disorders, number of chronic conditions, and household annual income were observed. In summary, this study provides preliminary insight into the relationships between types of self-care disability and depressive symptoms in middle-aged and older people.

The prevalence of depressive symptoms among elderly people in China is reported to range from 13–41% [34–36]. A national survey in China reported that approximately 30% of men and 43% of women aged 45 years and older experienced depressive symptoms [37]. In this study, the prevalence of depressive symptoms was 45.1%, which is similar to the results of previous studies among Chinese middle-aged and elderly people [38]. The prevalence of depressive symptoms in this study is higher than that reported in European (20.3%) [39] and Korean (12.4%) [40] samples using the CESD scale, but lower than that reported in samples from Brazil (45.77%) [41], Nepal (51.3%) [42], and Bangladesh (55%) [43] using the Geriatric Depression Scale (GDS-15). The variation in the prevalence of depressive symptoms may be related to differences in sample composition, assessment tools, and cut-off points in different studies [19].

Studies have shown that depressive symptoms among the elderly are related to many factors, including aging-related and disease-related processes, heredity factors, and psychosocial adversity-economic impoverishment [44]. There is increasing evidence indicating that ADL disability is related to depressive symptoms [45, 46]. Studies have shown that ADL disability may increase the risk of depressive symptoms in Chinese middle-aged and elderly people and their spouses [47]. In agreement with previous studies, the results of the current study provide support for the association between self-care disability and depressive symptoms, and among the influencing factors evaluated in the current study, self-care disability was the greatest risk factor for depressive symptoms. This suggests that middle-aged and elderly people with a self-care disability are at higher risk of depressive symptoms. In addition to self-care disability, the results indicated that female gender, age less than 60 years old, living in rural areas, widowed or separated, agricultural hukou, memory disorders, chronic diseases, and low- or middle household income were risk factors for depressive symptoms.

The results of this study also indicated that five types of self-care disability were risk factors for depressive symptoms, including dressing, bathing, transferring in and out of bed, using the toilet, and controlling urination and defecation. This may indicate that feeling disabled or dependent on others for these five types of daily self-care activities may lead to a decrease in self-esteem and an increased psychological burden, which may lead to a higher prevalence of depressive symptoms [48]. In other words, among middle-aged and elderly people with a self-care disability, we must pay more attention to those who specifically need assistance with dressing, bathing, transferring in and out of bed, using the toilet, and controlling urination and defecation, because they are at the highest risk of experiencing depressive symptoms. Furthermore, the results indicated that middle-aged and elderly Chinese people with difficulty controlling urination and defecation had the greatest risk of depressive symptoms. Difficulty controlling urination and defecation may affect an individual’s perceived dignity, thus and then affect contributing to depressive symptoms [49–51]. As such, there may be several mediating variables that can help explain the relationship between difficulty controlling urination and defecation and depressive symptoms. At the same time, this study did not find an association between difficulty eating and depressive symptoms after adjustment, which may be related to the smaller sample size. Only 247 people who had difficulty eating were included in this study, and the small sample size may cause bias in the results. Future studies should confirm the differential associations between depressive symptoms and different types of self-care disabilities and explore the mechanisms that may explain these differences. Further, more research is needed to examine the psychological changes and mental health of those who experience disability related to the five types of self-care activities.

In the current study, a positive correlation between the number of self-care disabilities and the risk of depressive symptoms was found, whereby each additional type of disability was associated with a greater than 2.6-fold increase in the risk of depressive symptoms. Moreover, an association between the quantity of self-care disabilities and the CESD-10 score was observed. Similarly, Xiang et al. suggested that there is a strong dose-response relationship between the number of disabilities and depression [11]. However, this previous study focused on a wider range of disabilities, including physical disability, communication disability, cognitive disability, and self-care disability. Therefore, the comprehensive results in the current study could provide useful information for the development of effective interventions and for improving the quality of daily living for patients with self-care disabilities. Future research should further explore the psychological changes and coping strategies of middle-aged and elderly people with varying numbers of self-care disabilities.

There are several limitations of this study that should be noted. First, self-report was the main method used to gain data on respondents’ depressive symptoms and self-care disability, and for respondents who required assistance expressing this information, this information was reported by the respondent’s caregivers, which could have introduced recall bias. Second, the CESD-10 scale was only used to measure negative feelings and behaviors during the past week. It is well known that depressive symptoms are not irreversible diseases. It is possible that for some respondents, depressive symptoms may have resolved or reduced a week ago. Although respondents who had received medication or psychotherapy were excluded from this study, there may have been some patients who experienced relief of symptoms without any external intervention. Therefore, the risk of depressive symptoms suggested by this study may be underestimated. Third, this study only provides evidence of an association between depressive symptoms and self-care disability; the true relationship between depressive symptoms and self-care disability requires evaluation via a cohort study.

## Conclusion

In conclusion, these findings reveal that individuals with a self-care disability have a significantly higher risk of depressive symptoms compared to healthy individuals. Individuals with a self-care disability related to dressing, bathing, transferring in and out of bed, using the toilet, and controlling urination and defecation have a significantly higher risk of depressive symptoms compared to those without these difficulties. Moreover, this study found a positive correlation between the number of self-care disabilities and depressive symptoms. In the future, a cohort study should be established to verify these conclusions.
